# Long-term surgical outcomes of pediatric cataract—multivariate analysis of prognostic factors

**DOI:** 10.1038/s41598-023-49166-2

**Published:** 2023-12-08

**Authors:** Tetsuro Oshika, Takao Endo, Daijiro Kurosaka, Naoko Matsuki, Mai Miyagi, Takafumi Mori, Toshiyuki Nagamoto, Kazuno Negishi, Sachiko Nishina, Koji Nomura, Noriyuki Unoki, Shigeo Yoshida

**Affiliations:** 1 Department of Ophthalmology, Faculty of Medicine, University of Tsukuba, 1-1-1 Tennoudai, Tsukuba, Ibaraki 305-8575 Japan; 2 Osaka Women’s and Children’s Hospital, Osaka, Japan; 3 Department of Ophthalmology, Iwate Medical University School of Medicine, Iwate, Japan; 4 Department of Ophthalmology, Kyorin University School of Medicine, Tokyo, Japan; 5 Aichi Children’s Health and Medical Center, Aichi, Japan; 6 Department of Ophthalmology, Fukushima Medical University, Fukushima, Japan; 7 Nagamoto Eye Clinic, Tokyo, Japan; 8 Department of Ophthalmology, Keio University School of Medicine, Tokyo, Japan; 9 National Center for Child Health and Development, Tokyo, Japan; 10 Hyogo Prefectural Kobe Children’s Hospital, Hyogo, Japan; 11 Osaka City General Hospital, Osaka, Japan; 12 Department of Ophthalmology, Kurume University School of Medicine, Fukuoka, Japan

**Keywords:** Medical research, Outcomes research, Paediatric research

## Abstract

We assessed the 10-year postoperative outcomes of pediatric cataract patients who underwent surgery at the age of 6 years or younger. A retrospective review of medical charts was conducted for 457 eyes of 277 patients, with the age at surgery averaging 1.3 ± 1.5 years (mean ± SD) and the follow-up duration averaging 12.8 ± 2.4 years (ranging from 10 to 17 years). The cohort included 250 eyes of 125 cases with bilateral aphakia (age at surgery 0.5 ± 0.8 years), 110 eyes of 55 cases with bilateral pseudophakia (1.9 ± 1.6 years), 42 cases with unilateral aphakia (1.1 ± 1.3 years), and 55 cases with unilateral pseudophakia (2.6 ± 1.7). A forward stepwise multiple regression analysis revealed that the best-corrected visual acuity at the final visit was significantly associated with laterality of cataract (with bilateral cases showing better results compared to unilateral cases), presence of systemic comorbidities, presence of ocular comorbidities, development of glaucoma, and phakic status (with better results in the pseudophakia group than the aphakia group). The age at surgery did not significantly affect visual acuity outcomes. A multiple logistic regression analysis demonstrated that the incidence of secondary glaucoma was significantly linked to younger age at surgery, phakic status (higher in aphakic than pseudophakic eyes), and presence of systemic comorbidities. In conclusion, after pediatric cataract surgery, final visual acuity was better in patients with bilateral cataracts, those treated with an intraocular lens, and cases without systemic or ocular comorbidities and secondary glaucoma. The development of secondary glaucoma was linked to younger age at surgery, aphakic status, and presence of systemic comorbidities.

## Introduction

Pediatric cataracts pose unique challenges distinct from adult cases^1–3^. Children present with a broader spectrum of clinical manifestations. Their cataracts may be either congenital or developmental, with varying degrees of amblyopia risk. The laterality of cataract, whether bilateral or unilateral, is also crucial, necessitating careful consideration of the optimal timing for surgical intervention. Given the ongoing growth of pediatric eyes, it is vital to account for each child's ocular developmental stage, along with any coexisting systemic and ocular comorbidities. Surgically, multiple options should be considered, such as the use of an intraocular lens (IOL) and its power, as well as strategies to manage the posterior capsule and anterior vitreous. Postoperatively, infantile eyes are prone to more pronounced inflammatory responses^1,2^. Prompt visual rehabilitation is essential, with an emphasis on amblyopia therapy and refractive adjustments. Furthermore, pediatric patients are at an elevated risk of complications, including visual axis opacification and secondary glaucoma.

Considering the immature nature of pediatric eyes and their continuous growth after surgery, long-term patient monitoring is mandatory. There is also a requisite to contemplate multitude factors when identifying prognostic indicators for this demographic, especially in light of the presence of various anatomical and physiological confounding variables. However, apart from a few studies centered on secondary glaucoma^4,5^, there is a paucity of research evaluating the long-term clinical courses of pseudophakic and aphakic eyes after childhood cataract surgery using multivariate analysis^6^. Our study was undertaken to assess the impact of various factors on the long-term outcomes of cataract surgeries in the pediatric population.

## Patients and methods

### Patients

We conducted a retrospective review of medical records from patients who underwent pediatric cataract surgery at eleven surgical sites across Japan. The inclusion criteria encompassed patients operated on at the age of 6 years or younger and who had a postoperative follow-up duration of at least 10 years. We excluded cases of traumatic cataract and cataracts secondary to other ocular diseases. Since it is challenging to clearly distinguish congenital and developmental cataracts, both clinical categories were considered for this research. While we included the eyes of infants with systemic and ocular comorbidities, those diagnosed with congenital glaucoma were excluded from our study. Those in the pseudophakic group underwent primary IOL implantation, and eyes that received secondary IOL implantation were not included in this study.

This study adhered to the principles set out in the Declaration of Helsinki. The institutional review board of each participating institute gave their approval for the study protocol. The committee at Tsukuba University Hospital determined that patient informed consent was not necessary for the use of their medical record data, in accordance with the Japanese Guidelines for Epidemiologic Study issued by the Japanese Government. Due to the study's observational nature, there was no requirement for clinical trial registration.

### Data collection and interpretation

Prior research has indicated that to minimize the effects of visual deprivation in congenital cataract, the ideal window for surgical intervention is within 6 weeks for unilateral cases and 10 weeks for bilateral cases^7–13^. This is because before these timeframes, vision loss from form deprivation is unlikely to take place, as the still-maturing visual system primarily depends on subcortical pathways. In our study, we evaluated the age of patients at the time of surgery in relation to these latent periods.

Glaucoma classification was based on previously established definitions for childhood glaucoma^14–16^. In summary, glaucoma was diagnosed using a combination of criteria, wich included intraocular pressure (IOP) exceeding 21 mm Hg, abnormal corneal enlargement greater than 11 mm, progressive increase in cup-disc ratio, cup-disc asymmetry of 0.2 when optic discs were of similar size, focal rim thinning, requirement for medication to lower IOP, and undergoing surgery specific to glaucoma. Suspected glaucoma was identified if the IOP was above 21 mm Hg, but other definitive criteria for glaucoma were absent. In instances where abnormal glaucomatous changes or increased IOP were observed, consultations with in-house glaucoma specialists were sought to ascertain the diagnosis of either glaucoma or suspected glaucoma.

The performance of any secondary surgical intervention was identified from the medical records, including procedures to clear visual axis opacification, either surgically or via laser capsulotomy, as well as glaucoma surgery and retinal detachment surgery. In determining the incidence of reoperation, procedures for strabismus were excluded.

### Statistical analysis

Numerical data are presented as mean ± standard deviation (SD) unless otherwise specified. For comparisons between two groups, the Mann–Whitney U-test was utilized. When assessing differences in numerical data across three or more groups, the Kruskal–Wallis test was employed, accompanied by a Bonferroni adjustment for multiple comparisons. The Pearson correlation test was applied to evaluate correlations between two parameters. Categorical data were analyzed using the chi-square test and Fisher’s exact test to assess incidence rates. A forward stepwise multiple regression analysis was conducted to identify independent variables associated with the best-corrected visual acuity at the final visit. To ascertain factors linked to the incidence of secondary glaucoma, a multiple logistic regression analysis employing a Wald selection criterion was utilized. All statistical tests were two-sided, and a *p*-value of ≤ 0.05 was considered indicative of statistical significance. Analyses were conducted using SPSS software version 29 (IBM Corp, Armonk, NY).

## Results

**Figure 1:**
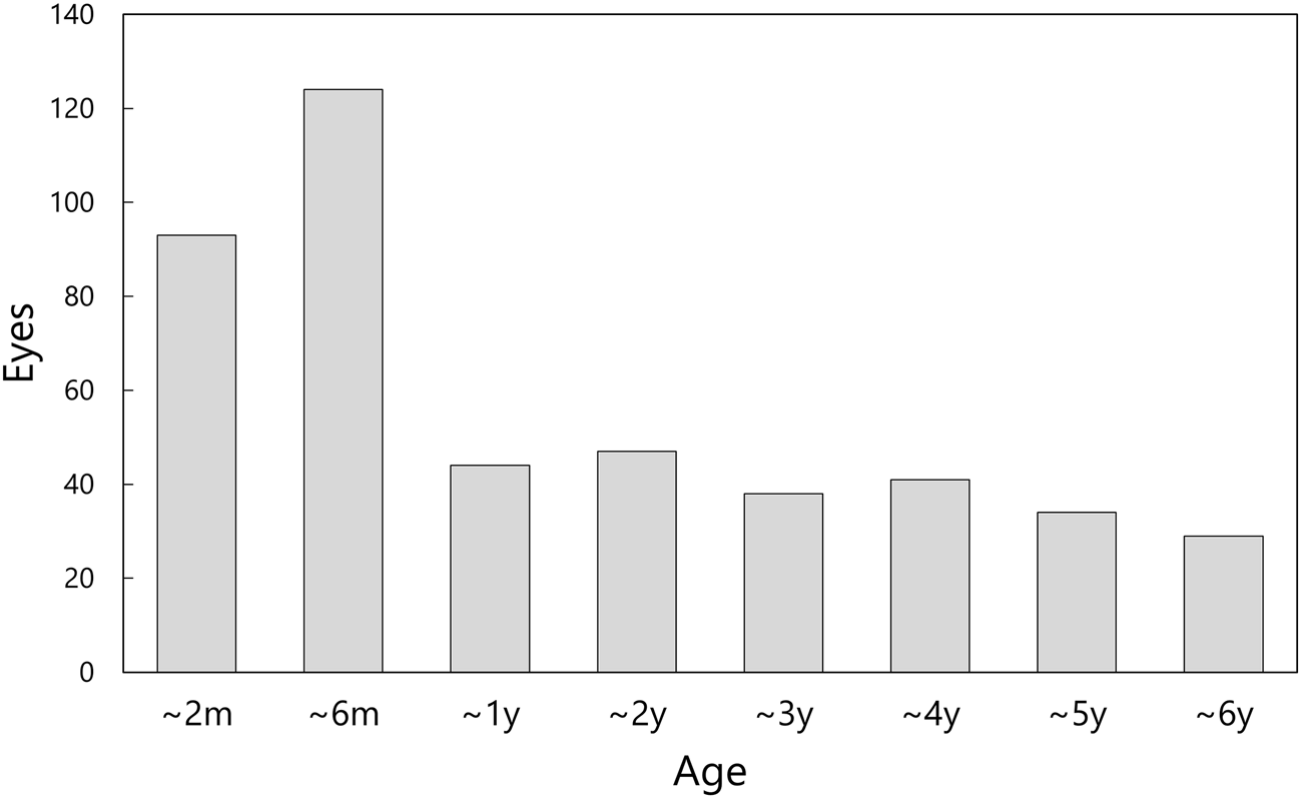
Distribution of patient’s age at the time of cataract surgery.

A total of 457 eyes of 277 patients met the inclusion criteria and their medical records were analyzed. The mean age at the time of cataract surgery was 1.3 ± 1.5 years, ranging from 1 month to 6 years, with its distribution depicted in Fig. 1. The mean follow-up duration was 12.8 ± 2.4 years, spanning 10–17 years.

Of these patients, 180 cases had bilateral cataract and 97 cases had unilateral cataract. The average age at the time of surgery for bilateral and unilateral patients was 0.9 ± 1.3 years and 2.0 ± 1.7 years, respectively, with a significant difference between them (*p* < 0.001). In relation to the latent periods critical for visual system development, 16% (29/180) of the bilateral cataract patients underwent surgery within the first 10 weeks after birth and 9% (9/97) of the unilateral cataract patients received surgery within the initial 6 weeks; the difference was not statistically significant (*p* = 0.143).

The subsequent analyses were conducted for the following four categories; 250 eyes of 125 cases with bilateral aphakia (age at surgery; 0.5 ± 0.8 years), 110 eyes of 55 cases with bilateral pseudophakia (1.9 ± 1.6 years), 42 eyes of 42 cases with unilateral aphakia (1.1 ± 1.3 years), and 55 eyes of 55 cases with unilateral pseudophakia (2.6 ± 1.7). Significant age differences at surgery were observed between the bilateral aphakia and bilateral pseudophakia groups (*p* < 0.001), between the bilateral aphakia and unilateral aphakia groups (*p* < 0.001), and between the unilateral aphakia and unilateral pseudophakia groups (*p* < 0.001). No significant difference was noted between the bilateral pseudophakia and unilateral pseudophakia groups (*p* = 0.758). Systemic and ocular comorbidities identified before surgery for each group are summarized in Table 1.

**Table 1:** Patients’ demographics and preoperative comorbidities.

	Bilateral patients		Unilateral patients	
	Aphakia	IOL	Aphakia	IOL
Number of eyes (patients)	250 (125)	110 (55)	42 (42)	55 (55)
Age at the time of surgery (years)	0.5 ± 0.8	1.9 ± 1.6	1.1 ± 1.3	2.6 ± 1.7
Trisomy 21 and other congenital abnormalities	15 (6.0%)	4 (3.6%)	3 (7.1%)	3 (5.5%)
Cardiovascular disorders	4 (1.6%)	6 (5.5%)	3 (7.1%)	2 (3.6%)
Microcornea	22 (8.8%)	8 (7.3%)	3 (7.1%)	2 (3.6%)
Microphthalmia	13 (5.2%)	4 (3.6%)	3 (7.1%)	1 (1.8%)
Lens hypoplasia	2 (0.8%)	6 (5.5%)	1 (2.4%)	1 (1.8%)
Persistent fetal vasculature	2 (0.8%)	0	4 (9.5%)	1 (1.8%)

Number of eye (percentage).

**Figure 2:**
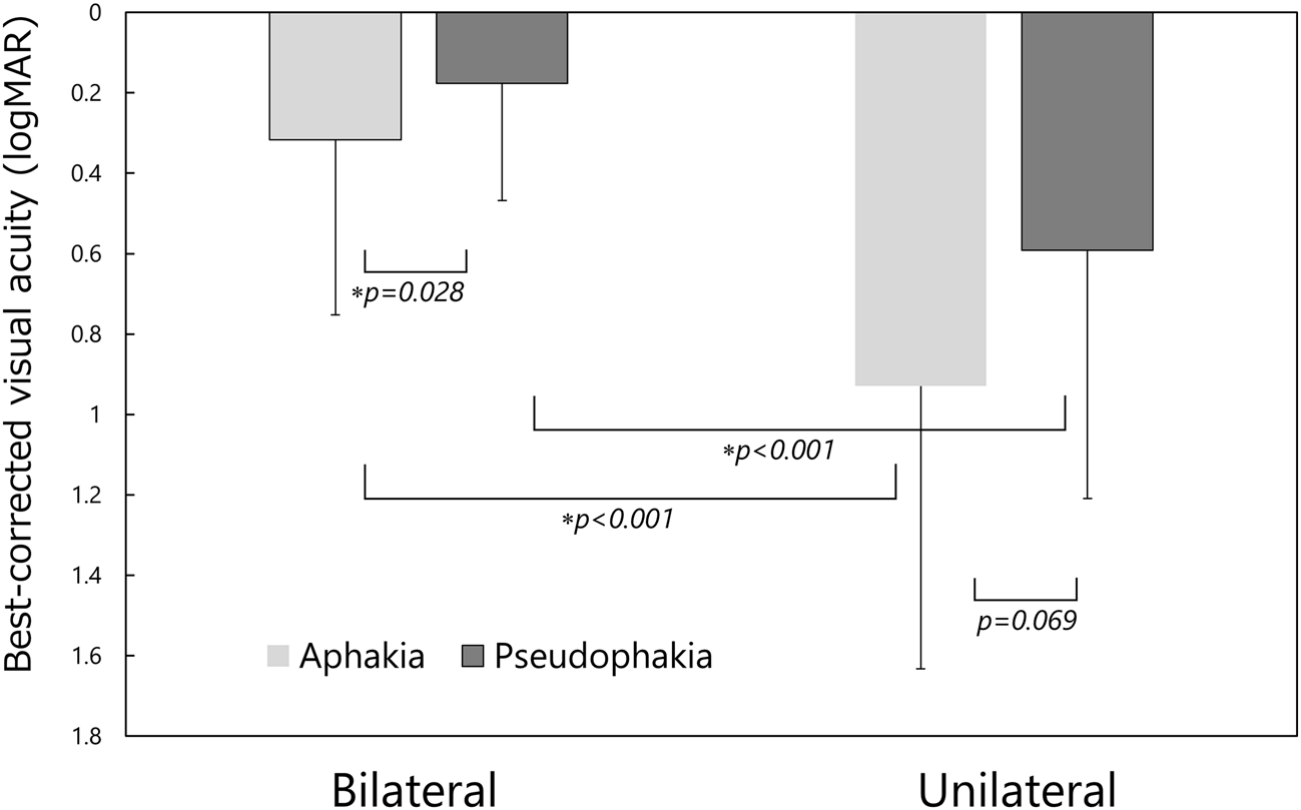
Best-corrected visual acuity at the final visit. *Kruskal–Wallis test with Bonferroni adjustment.

The overall mean best-corrected visual acuity at the final visit was 0.404 ± 0.579 (logMAR), which did not correlate with the patient’s age at the time of surgery (r =  − 0.087, *p* = 0.071). The logMAR values at the final visit were 0.317 ± 0.435 for bilateral aphakic cases, 0.176 ± 0.292 for bilateral pseudophakia cases, 0.929 ± 0.703 for unilateral aphakic cases, and 0.592 ± 0.617 for unilateral pseudophakia cases. Statistical comparisons among these groups are illustrated in Fig. 2.

Postoperative complications are summarized in Table 2. The incidence of secondary glaucoma was significantly higher in the aphakia groups compared to the pseudophakia groups, both in the bilateral and unilateral cases. Conversely, the rate of visual axis opacification was significantly higher in the pseudophakia groups than in the aphakia groups. The incidence of retinal detachment was not different between the aphakia and pseudophakia groups.

**Table 2:** Postoperative complications.

	Bilateral patients			Unilateral patients		
	Aphakia	IOL		Aphakia	IOL	
Postoperative complications						
Glaucoma	40 (16.0%)	1 (0.9%)	*p* < 0.001	6 (14.3%)	0	*p* = 0.005
Suspected glaucoma	7 (2.8%)	4 (3.6%)	*p* = 0.742	0	2 (3.6%)	*p* = 0.504
Visual axis opacification	12 (4.8%)	26 (23.6%)	*p* < 0.001	0	10 (18.2%)	*p* = 0.004
Retinal detachment	4 (1.6%)	1 (0.9%)	*p* = 1.000	0	1 (1.8%)	*p* = 0.504
Secondary surgical interventions						
Glaucoma	12 (4.8%)	1 (0.9%)	*p* = 0.120	1 (2.4%)	0	*p* = 0.433
Visual axis opacification	13 (5.2%)	24 (21.8%)	*p* < 0.001	0	12 (21.8%)	*p* < 0.001
Retinal detachment	4 (1.6%)	1 (0.9%)	*p* = 1.000	0	1 (1.8%)	*p* = 1.000
IOL repositioning	–	1 (0.9%)	–	–	1 (1.8%)	-

Number of eyes (percentage).

The incidence of secondary surgical intervention is also showcased in Table 2. A higher number of pseudophakic patients underwent procedures to clean visual axis opacification both in the bilateral and unilateral surgery groups. IOL repositioning surgery was conducted in two cases; one case each from the bilateral and unilateral pseudophakic eye groups.

A forward stepwise multiple regression analysis revealed that the best-corrected visual acuity at the final visit was significantly associated with several independent variables as shown in Table 3. These included laterality (bilateral eyes showed superior results compared to unilateral eyes), presence of systemic comorbidities, presence of ocular comorbidities, development of glaucoma, and phakic status (the pseudophakia group outperforming the aphakia group). Factors that were not significant and excluded from the regression model were patient’s age at surgery, development of visual axis opacification, retinal detachment, whether anterior vitrectomy was performed during the initial cataract surgery, and the surgical sites.

**Table 3:** Variables influencing best-corrected visual acuity at the final visit: A forward stepwise multiple regression analysis.

Variables in the regression model	Standardized partial regression coefficient	
Laterality (bilateral > unilateral)	− 0.375	*p* < 0.001
Systemic comorbidities	0.211	*p* < 0.001
Ocular comorbidities	0.174	*p* < 0.001
Glaucoma	0.108	*p* = 0.016
Phakic status (IOL > aphakia)	0.107	*p* = 0.017
Excluded variables		
Age at surgery		*p* = 0.609
Visual axis opacification		*p* = 0.877
Retinal detachment		*p* = 0.994
Performance of primary anterior vitrectomy		*p* = 0.861
Facilities		*p* = 0.177 ~ 0.918

A multiple logistic regression analysis demonstrated that the occurrence of secondary glaucoma was linked to age at surgery, phakic status (with aphakic eyes having a higher incidence than pseudophakic eyes), and presence of systemic comorbidities (Table 4). The laterality of cataract, existence of ocular comorbidities, performance of anterior vitrectomy during the primary cataract surgery, and the surgical sites were found to be insignificant.

**Table 4:** Variables influencing glaucoma development: A Wald selection multiple logistic regression analysis.

Variables in the regression model	Odds ratio, exp (b)	
Age at surgery	0.934	*p* = 0.008
Phakic status (aphakia > IOL)	10.889	*p* = 0.021
Systemic comorbidities	2.935	*p* < 0.001
Excluded variables		
Laterality (bilateral or unilateral)		*p* = 0.690
Ocular comorbidities		*p* = 0.333
Performance of primary anterior vitrectomy		*p* = 0.435
Facilities		*p* = 0.190 ~ 0.857

The incidence of secondary surgical interventions was also assessed using a multiple logistic regression analysis. Factors that were independently associated with the need for reoperation included younger age at the time of surgery (*p* < 0.001), pseudophakic status (*p* < 0.0001), presence of ocular comorbidities (*p* = 0.003), and the absence of anterior vitrectomy during the initial cataract surgery (*p* < 0.001).

## Discussion

In our study, we employed multivariate analysis to evaluate the long-term outcomes of pediatric cataract surgery. The multiple regression analysis indicated that pseudophakic eyes achieved better final visual acuity than aphakic eyes, and bilateral cataract patients had more favorable visual acuity outcomes than unilateral cataract patients. Repka et al^17^. found in their evaluation of 5 year ophthalmic outcomes after cataract surgery in children under 13 years that the median visual acuity was 20/63 in bilateral aphakic eyes, 20/32 in bilateral pseudophakic eyes, 20/200 in unilateral aphakic eyes, and 20/65 in unilateral pseudophakic eyes. Solebo et al^18^., in their assessment of results following primary IOL implantation in children aged two and under with congenital or infantile cataract, showed a median visual acuity at 5 years of 0.34 logMAR for bilateral cataract and 0.70 logMAR in the operated eye for unilateral cataract. Vasavada et al^19^. examined visual outcomes following bilateral congenital cataract surgery in children up to 2 years of age and noted that, at a 5 year follow-up, the mean logMAR visual acuity was 0.59 ± 0.33 in the aphakia group and 0.5 ± 0.23 in the pseudophakia group. Notably, eyes in the pseudophakia group began presenting documentable vision earlier in their postoperative evaluations. Rajavi et al^20^., in their study involving patients with an average age of 65 ± 66.6 months at the time of surgery and a surgical interval of 12.9 ± 23.5 months, observed that pseudophakic eyes achieved mean best-corrected visual acuity of 0.29 ± 0.28 logMAR, while aphakic eyes reached 0.70 ± 0.53 logMAR, indicating that unilateral cataract and aphakia are potential risk factors for suboptimal visual acuity. Our results align with these previous studies, reaffirming the effectiveness of contemporary IOL implantation surgery for pediatric patients and highlighting the challenges in obtaining satisfactory visual outcomes in cases of unilateral infantile cataract.

In our study, we found that the presence of systemic and ocular comorbidities, as well as the development of secondary glaucoma, were independent variables adversely impacting the final visual acuity. Past reviews have identified these parameters as risk factors for poor visual prognosis in eyes affected by congenital and developmental cataracts^1–3^. While our study included patients with preexisting systemic or ocular abnormalities, it is possible that those with severe abnormalities discontinued their visits, resulting in a loss to follow-up. Our study protocol, however, wasn’t tailored to capture data on such cases. Nonetheless, systemic and ocular complications were found to be negative prognostic indicators in our analysis.

The results of our multiple logistic regression analysis demonstrated that the occurrence of secondary glaucoma was significantly associated with phakic status, with aphakic eyes having a higher incidence than pseudophakic eyes. Recent reports utilizing modern surgical techniques have shown a reduced incidence of glaucoma in pseudophakic eyes, indicating that primary IOL implantation combined with anterior vitrectomy does not increase the risk of secondary glaucoma^17,21–23^. In addition, other large-scale analyses have highlighted that primary IOL implantation serves as a protective factor against glaucoma development after congenital cataract surgery^24–27^. A 10-year follow-up study of IOL implantation surgery in pediatric patients demonstrated a significantly low rate of glaucoma^28^. On the other hand, a randomized controlled trial of cataract surgery in infants, comparing cases with and without IOL implantation, did not find a difference in the rate of glaucoma in pseudophakic and aphakic eyes after a 10-year follow-up^29^. A retrospective study like ours may be subjected to inherent biases, one of the limitations of the current study.

To rationalize how primary IOL implantation in pediatric cataract patients might reduce glaucoma incidence, two hypotheses have been proposed^24,27^. The first, the chemical theory, conjectures that harmful vitreous chemical components might damage the trabecular meshwork in an aphakic eye, while the presence of an IOL in a pseudophakic eye could limit or lessen this exposure. The second, the mechanical theory, suggests that the loss of support to the trabecular meshwork in aphakia could lead to its disorganization or even collapse, thereby compromising its function both as a filter and an active metabolic tissue. Implanting a posterior chamber IOL at the time of cataract removal might counteract this loss of support. The validity of these theories, however, remains a subject for future investigations.

Patient’s age at surgery was also an independent factor influencing the development of glaucoma. Numerous studies have indicated that younger patients at surgery are more prone to secondary glaucoma^6,26,30^, and several theories have been suggested to explain this^31^. First, underdeveloped anterior chamber angle in infants may lead to increased intolerance to surgery. Second, younger patients typically experience more intense postoperative inflammation, leading to greater exposure of the immature trabecular meshwork to a higher concentration of inflammatory cells and cytokines. Third, the performance of posterior capsulorrhexis and anterior vitrectomy may increase glaucoma risk by enhancing the interaction between the vitreous and trabecular meshwork. This third hypothesis, however, was not supported by the current multiple logistic analysis, where the performance of anterior vitrectomy was found to be an insignificant factor. Despite the increased risk of secondary glaucoma in younger patients, postponing cataract surgery is not advisable when considering the serious effects of visual deprivation. The potential risks of early cataract surgery should be weighed against minimizing the duration of visual deprivation, especially considering the latent periods for visual system development.

This study presents several limitations. Firstly, being a retrospective, multicenter investigation, there was no standardized approach to surgical methods and IOL indications. Secondly, pinpointing the precise age of onset for childhood cataract is challenging, leading us to include both congenital and developmental cataracts in our sample. Thirdly, the absence of newborn eye screening in our country makes it difficult to detect true congenital cataracts at a very early stage, resulting in varying durations between the onset of congenital cataracts and the timing of surgery among cases. Fourthly, our evaluation was limited to those who completed a postoperative follow-up of 10 years and longer. Information on participants who dropped out or were untraceable during the study is lacking. It is possible that those experiencing less favorable outcomes may have discontinued their regular visits. Fifthly, the specifics of postoperative amblyopia therapy were not analyzed in this study.

In conclusion, we conducted a retrospective analysis of 10-year outcomes after pediatric cataract surgery. Our multiple stepwise regression analysis revealed that the influential variables for the final best-corrected visual acuity were the cataract's laterality (with bilateral cases showing superior results to unilateral ones), the presence of systemic and ocular comorbidities, glaucoma development, and phakic status—with the pseudophakic group offering better results than the aphakic group. Additionally, our multiple logistic regression analysis indicated that the development of secondary glaucoma was significantly linked to the patient's age at surgery, phakic status (with aphakic eyes being more susceptible than pseudophakic eyes), and the presence of systemic comorbidities.

## Data Availability

The datasets generated during and/or analyzed during the current study are available from the corresponding author on reasonable request.
